# Determination of Curdlan Oligosaccharides with High-Performance Anion Exchange Chromatography with Pulsed Amperometric Detection

**DOI:** 10.1155/2018/3980814

**Published:** 2018-09-02

**Authors:** Lidong Cao, Huifang Tian, Miaomiao Wu, Hongjun Zhang, Puguo Zhou, Qiliang Huang

**Affiliations:** ^1^Institute of Plant Protection, Chinese Academy of Agricultural Sciences, No. 2 Yuanmingyuan West Road, Beijing 100193, China; ^2^Ministry of Agriculture, Institute for the Control of Agrochemicals, No. 22 Maizidian Street, Beijing 110000, China; ^3^Key laboratory of Carcinogenesis and Translational Research (Ministry of Education/Beijing), Central Laboratory, Peking University Cancer Hospital & Institute, No. 52 Fucheng Road, Beijing 100142, China

## Abstract

The increasing interest of curdlan oligosaccharides (COS) in medicine and plant protection fields implies a necessity to identify and quantify this product. In the present study, an efficient and sensitive analytical method based on high-performance anion exchange chromatography with pulsed amperometric detection (HPAEC-PAD) was established for the simultaneous separation and determination of D-glucose and *ß*-1,3-linked COS ranging from (COS)_2_ to (COS)_6_ within 20 min. Detection limits were 0.01 to 0.03 mg/L. The optimized assay was performed on a CarboPac-PA100 analytical column (4 mm × 250 mm) using isocratic elution with water−0.2 M sodium hydroxide−0.5 M sodium acetate mixture (50 : 30 : 20, v/v/v) as the mobile phase at a flow rate of 0.8 mL/min. Regression equations indicated a good linear relationship (*R*^2^ = 0.9992–1.0000, *n* = 6) within the test ranges. Quality parameters including precision and accuracy were fully validated and found to be satisfactory. More important, the regression of natural logarithm values of retention times (log_10_ RT) versus the degree polymerization (DP), as well as the slope coefficient of each COS's linear equation versus the corresponding DP, fitted a linear relationship well. These inherent linear relationships could provide valuable information to tentatively identify and quantify the COS even with the DP more than 6 without authentic standard. Furthermore, when the log_10_ RT was plotted against log_10_ flow rate for each COS, a perfect linear relationship was also observed.

## 1. Introduction

**Figure 1:**
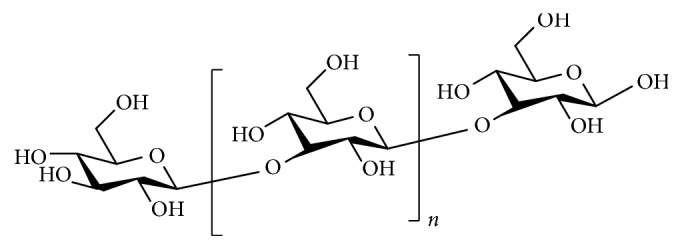
Chemical structure of curdlan.

Curdlan, which is industrially manufactured as an exopolysaccharide of bacterium, is the only linear *β*-(1,3)-glucan homopolysaccharide without branching (Figure 1) [1–3]. This biopolymer is soluble in alkaline solutions and dimethylsulphoxide [4], but insoluble in water and most organic solvents due to the unique gel properties [5], which limits its wide biological utility and industrial applications. As a consequence, curdlan has traditionally been used as a stabilizer, texturizer, and thickener in the food industry [6]. In recent decades, the study on curdlan has attracted interest in converting it into more soluble curdlan oligosaccharides (COS). COS are produced mainly by acidic [7–10] or enzymatic [8, 11–14] hydrolysis, hydrogen peroxide-induced oxidative degradation [15], ultrasonication [16], and microwave-assisted hydrothermal hydrolysis [17].

These hydrolyzed oligomers of curdlan exhibit remarkable biomedical functions, such as antitumor and immunological activities [18, 19]. Furthermore, COS can also play an efficient role in plant protection and improvement for sustainable agriculture since it can efficiently activate the plant innate immune defense system prior to the infection of pathogens [20]. For instance, COS can elicit the plant's natural defense responses in tobacco (*Nicotiana tabacum* L.) [21], potato (*Solanum tuberosum* L. cv. McCain G1) [22], and *Arabidopsis thaliana* Col0 [23] against tobacco mosaic virus, *Phytophthora infestans*, and *Botrytis cinerea* infections, respectively. It is well accepted that the physiochemical properties and biological activities of COS are closely related to and dependent on the chemical structure and degree of polymerization (DP). Therefore, it is necessary to establish a highly sensitive and selective method for quality control of COS, including DP and single COS content determination.

For qualitative and quantitative analysis of COS, various methods have been developed. Clarke et al. developed a paper chromatography method to analyze the COS from lichenase-hydrolyzed barley *β*-glucan, but this technique is time-consuming (approximately 47 h) [24]. For detailed structural information, matrix-assisted laser desorption/ionization time-of-flight mass spectrometry (MALDI-TOF-MS) is frequently used for rapid qualitative analysis of COS [12, 25]. For separation and analysis of COS with different DPs, chromatography coupled with various detectors can provide rapid preliminary information of DP, especially for their quantitative analysis. High-performance liquid chromatography (HPLC) coupled with a refractive index (RI) [10, 12, 22] and evaporative light-scattering detector (ELSD) [25] are always employed. However, separation by HPLC and detection by RI and ELSD have their inherent limitation, such as poor resolution and low detection sensitivity. Therefore, availability of a simple method for detecting COS is still needed. High-performance anion exchange chromatography (HPAEC) combined with pulsed amperometric detection (PAD) is a powerful tool for an efficient separation and highly sensitive detection for carbohydrate without the need for hydrolysis or further derivatization [26, 27]. HPAEC-PAD for separating COS has previously been reported [25, 28–31]. However, most of these methods are only used for qualitative analysis to monitor the hydrolysis process of curdlan. None of these HPAEC-PAD methods for assaying D-glucose and *β*-1,3-linked COS with the DPs 2–6 ((COS)_2–6_) was fully validated. In addition, no detailed chromatographic behaviors were studied for COS.

In the present study, a HPAEC-PAD method for separating and detecting D-glucose and (COS)_2-6_ was developed. Quality parameters such as sensitivity, linearity, precision, and accuracy were fully validated. Moreover, the linear relationship between the retention time of COS and its corresponding DP, as well as the linear relationship between the detector response of COS and its corresponding DP, was established, which could provide valuable information to identify and quantify the COS even with the DP more than 6.

## 2. Experimental Section

### 2.1. Materials

Five COS standards with the DPs 2–6 were purchased from Megazyme, and all the structures were further verified by MALDI-TOF-MS. D-glucose (≥98%) was obtained from J&K Scientific Ltd. Sodium hydroxide solution (50%, w/w, used for mobile phase preparation) was purchased from Alfa Aesar China Co., Ltd., and sodium acetate (99%) was obtained from Sigma Aldrich Co. LLC. Primary stock solutions at concentration of about 100 mg/L were prepared by dissolving D-glucose and each single COS standard, respectively, using deionized water, which was obtained using a MilliQ (Millipore, Bedford, MA, USA) water purification system. Working standard solutions were prepared as needed by dilution of the stock solutions with water.

### 2.2. HPAEC-PAD Analysis

Chromatographic analysis was conducted on an ICS-3000 system (Dionex, Sunnyvale, CA, USA) equipped with a CarboPac PA-100 guard column (4 mm × 50 mm) and a CarboPac-PA100 analytical column (4 mm × 250 mm). D-glucose and COS were detected by pulsed amperometric detection (PAD) with a gold working electrode and an Ag/AgCl reference electrode using a standard carbohydrate quadruple potential waveform. Data processing was performed using a Chromeleon 6.8 chromatogram workstation.

Isocratic elution was optimized by employing water (eluent A), 0.2 M aqueous sodium hydroxide (eluent B), and 0.5 M sodium acetate solution (eluent C). Eluent B was prepared according to our previously developed HPAEC-PAD method for the determination of chitooligosaccharides [32]. To prepare eluent C, dispense approximately 800 mL of water into a 1 L plastic bottle, add a stir bar and begin stirring, and add 41.0 g of anhydrous sodium acetate steadily to the briskly stirring water to avoid the formation of clumps. After the salt dissolves, remove the stir bar with a magnetic retriever, and the mixture was diluted up to the 1 L line with water. All mobile phases were degassed and pressurized with high purity nitrogen to inhibit adsorption of carbon dioxide and subsequent production bicarbonate contamination, which would change the composition of eluent and shorten the retention time. Moreover, all the mobile phase could not be used beyond one week. Always use the same methodology for eluent preparation to ensure consistency. The elution program was performed at a flow rate of 0.8 mL/min, and 25 *μ*L was injected.

### 2.3. Calibration

To assess linearity, calibration curves were plotted by the partial least squares method on the analytical data of peak area and concentration, using analyte standards covering the concentration range of 0.3–10.5 mg/L for D-glucose, 0.3–9.5 mg/L for (COS)_2_, 0.4–11.4 mg/L for (COS)_3_, 0.3–10.7 mg/L for (COS)_4_, 0.3–9.7 mg/L for (COS)_5_, and 0.4–11.4 mg/L for (COS)_6_. Six equispaced concentrations were chosen for each COS, and duplicated injections were performed at each level. All analyses were carried out in duplicate. The dilute standard solution was further diluted to the known low concentration with water for signal-to-noise (S/N) ratio determination. The limits of detection (LOD) and quantification (LOQ) were determined as the lowest concentrations generating S/N ratios of 3 and 10, respectively.

### 2.4. Method Validation

The precision of the method was assessed according to the repeatability (intraday) and intermediate (interday) precision, which was expressed as relative standard deviation (%RSD). For the mixed D-glucose and each single COS standard solution, the precision of the method in the aspect of retention time and peak area was determined. Repeatability was evaluated with seven replicates in one single HPEAC run. Intermediate precision was assessed using nine determinations (three determinations daily over three days) using the same equipment, but performed on three consecutive days using three separately prepared batches of eluent. Both repeatability and intermediate precision were performed at three (low, middle, and high) concentrations levels within the linear range.

The accuracy of the method was determined by standard addition-recovery experiment. A known amount of the each working standard solution was added to the blank solution, and the prepared spiked sample was analyzed. The concentration of each analyte was calculated from the corresponding calibration curve that gave the relationship between the amount of analyte and the peak area, and recovery was calculated using the following formula: recovery (%) = (observed amount − original amount)/spiked amount × 100%. Three different concentrations for each COS were spiked, and triplicate determinations were carried out for each COS standard addition.

## 3. Results and Discussion

### 3.1. Optimization and Chromatographic Behaviors

**Figure 2:**
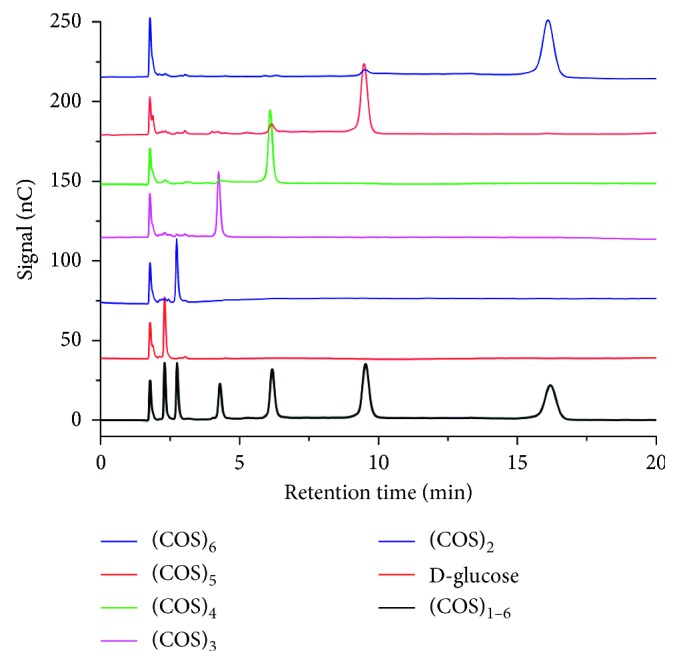
Typical HPAEC-PAD chromatograms of the mixed (COS)_1–6_ standards on a CarboPacPA-100 column.

When applied for carbohydrates, HPAEC-PAD affords high-resolution separation and sensitive detection, which has been extensively used in carbohydrates analysis. Native COS is a suitable candidate for this well-accepted analytical method. Satisfactory chromatographic separation mainly depends on the column, mobile phase composition, and flow rate. These variables were optimized using mixed D-glucose and (COS)_2–6_ standard solutions. Results indicated that the column of CarboPac-PA100 delivered a better separation than CarboPac-PA10. The mobile phase composition and flow rate were screened, and results showed that isocratic elution with eluent A (water), B (0.2 M sodium hydroxide), and C (0.5 M sodium acetate) mixture (50 : 30 : 20, v/v/v) at 0.8 mL/min afforded approving baseline separation for (COS)_2–6_ within 18 min, as shown in Figure 2. Results indicated that when the volume of sodium acetate was constant, COS were eluted slower with the increasing volume of sodium hydroxide, and this is opposite to sodium acetate when the volume of sodium hydroxide was fixed, namely, the more ratio of sodium acetate, the faster elution of COS. Under each condition, the COS with the lowest DP eluted first, followed by COS with a higher DP. These elution patterns are in accordance with those obtained for normal homogenous oligosaccharides in ion chromatography, where retention times tend to increase with increasing DP [33].

**Figure 3:**
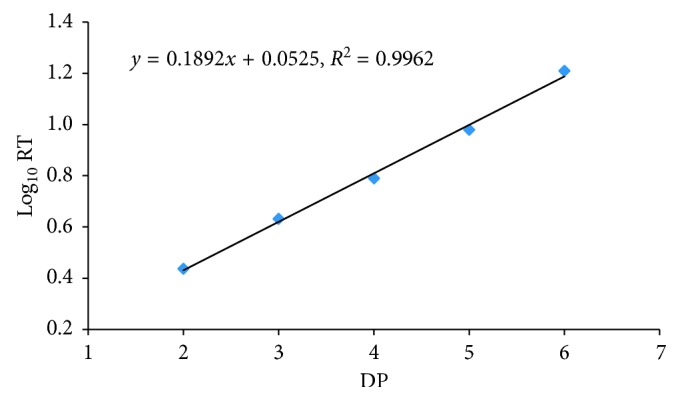
Linear relationship between log_10_ RT and DP for COS separated by HPAEC on a CarboPacPA-100 column using isocratic elution with eluent A (water), B (0.2 M sodium hydroxide), and C (0.5 M sodium acetate) mixture (50 : 30 : 20, v/v/v) at 0.8 mL/min.

Moreover, it is interesting that when the natural logarithm values of retention times (log_10_ RT) were plotted against DPs (2–6), a well linear relationship was observed as shown in Figure 3. This linear relationship is different from that reported for laminarin oligosaccharides [31]. In that report, log_10_ values of DP fitted a linear relationship with retention time. Due to the difficulty to obtain the standard of COS with higher DP, this inherent linear relationship is very useful for retention time prediction for those COS with DP beyond 6. Using this equation (*y* = 0.1892*x* + 0.0525, *R*^2^ = 0.9962, *y* standards for log_10_ RT, *x* standards for DP), the hypothetical retention times of (COS)_7_ and (COS)_8_ were estimated and were 23.8 and 36.8 min, respectively, which could provide guidance for the setting of total eluent time and chromatographic optimization, especially for tentative identification of the COS without authentic standard. Moreover, this interesting linear relationship is ubiquitous. When the chromatographic conditions such as the mobile phase compositions and flow rate changed, the regression of log_10_ RT versus the DP of the separated COS can still fit a linear relationship well, as presented in Table 1.

Another noticeable linear relationship was observed between the retention time and flow rate for each single COS. Five flow rates of 0.2, 0.4, 0.6, 0.8, and 1.0 mL/min were tested, the log_10_ RT was plotted against log_10_ flow rate (log_10_ FR) for each COS, and a perfect linear relationship was observed as presented in Table 2.

### 3.2. Calibration and Method Validation

**Figure 4:**
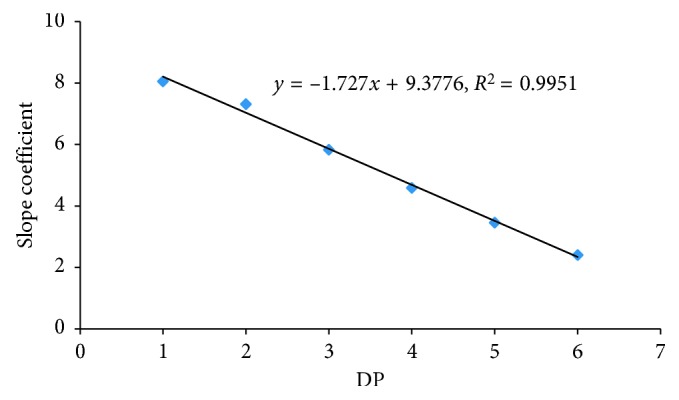
Linear relationship between the slope coefficient of each COS's regression equation and the corresponding DP.

Quality parameters such as sensitivity, linearity, precision, and accuracy were fully investigated. Results indicated that the linear ranges for (COS)_2–6_ were 0.3–10.5 mg/L. All the calibration curves showed good linearity (*R*^2^ = 0.9995–1.0000) in the tested range (Table 3). Slope coefficients of the linear regression equation, which are directly related to the detector response, gradually decreased with increasing DP. These response patterns on a weight basis were similar to what was observed for maltosaccharides and inulooligosaccharides with DPs 3–7, reported by Koch et al. [34] and Borromei et al. [35]. This could be reasonably interpreted by the decrease in molarity of the repeated unit with increasing DP at the same mass concentration. More important, we found that the regression of the slope coefficient of each COS's linear equation on the corresponding DP fitted a linear relationship well described in Figure 4. This inherent rule can be beneficial for semiquantification of COS without authentic standard using another COS standard with known DP.

The LOD and LOQ were defined as the minimum amounts at which the analyte can be reliably detected and quantified. Typical signal-to-noise (S/N) ratios of the LOD and LOQ were 3 and 10, respectively. Diluted low concentrations of the D-glucose and (COS)_2–6_ standard solutions were injected to determine the S/N ratio. Then the LOD and LOQ were calculated. In the present study, the LOD and LOQ for (COS)_2–6_ ranged from 0.01 to 0.03 and from 0.03 to 0.11 mg/L, respectively.

Precision was assessed by assaying known concentrations of the mixed D-glucose and (COS)_2–6_ standards. For repeatability (intraday) and intermediate (interday) precision to be established, variations in terms of peak areas and retention times at three concentration levels were determined (Table 4). Repeatability was determined with seven replicates in one day at three concentration levels. Under repeatability conditions, peak areas and retention times for all tested analytes were stable with 1.5–4.0 and 0.1–0.4% RSD, respectively. Intermediate precision was performed from nine determinations (three determinations daily over three days) with the same equipment. However, these determinations were conducted on three consecutive days using three separately prepared batches of eluents. Under intermediate precision conditions, peak areas and retention times for all tested analytes were stable with 2.5–5.1 and 0.4–1.3% RSD, respectively. The standard addition method was used to assess the method accuracy, and the recoveries were found to be acceptable ranging from 90.1% to 105.2% under three spiked concentration levels (Table 5). These validation results demonstrate that this HPAEC-PAD method is sensitive, precise, and accurate for the simultaneous quantitative determination of D-glucose and *ß*-1,3-linked COS with the DPs of 2–6.

## 4. Conclusions

The increasing interest of COS in medicine and plant protection fields implies a necessity to identify and quantify this product for structure-function relationship study. In the present work, a sensitive, efficient, and quick HPAEC-PAD method was developed and demonstrated to be suitable for separating, identifying, and quantifying D-glucose and *ß*-1,3-linked (COS)_2–6_ within 20 min. High sensitivity, satisfactory linearity, precision, and accuracy were obtained. Moreover, detailed chromatographic patterns were studied for D-glucose and COS. The inherent linear relationships for the retention time of each COS, as well as the detector response with the corresponding DP, were clearly observed. These linear relationships could provide valuable information to identify and quantify the COS even with the DP more than 6. Furthermore, a noticeable linear relationship was also observed between the retention time and flow rate for D-glucose and each single COS, which is helpful for chromatographic optimization. These interesting linear relationships could probably exist in other homogenous oligosaccharides with HPAEC-PAD determinations and find wide applications in both analytical and biological researches.

## Figures and Tables

**Table 1:** Linearity of the log_10_ RT versus DPs (2–6) of COS separated by HPAEC on a CarboPacPA-100 column.

Entry	Mobile phase (A : B : C)^a^	Flow rate (mL/min)	Linear equation^b^	*R* ^2^
1	40 : 40 : 20	0.5	*y* = 0.2183*x* + 0.2208	0.9964
2	40 : 40 : 20	0.8	*y* = 0.2204*x* + 0.0137	0.9963
3	40 : 40 : 20	1.0	*y* = 0.2199*x* − 0.0811	0.9964
4	30 : 50 : 20	0.8	*y* = 0.2408*x* − 0.0104	0.9964
5	50 : 30 : 20	0.8	*y* = 0.1892*x* + 0.0525	0.9962
6	35 : 40 : 25	0.8	*y* = 0.1597*x* + 0.0839	0.9942
7	45 : 40 : 15	0.8	*y* = 0.2949*x* − 0.0520	0.9981

^a^A: water; B: 0.2 M sodium hydroxide; C: 0.5 M sodium acetate; ^b^*y* and *x* refer to the log_10_ RT and DP, respectively.

**Table 2:** Linearity of the log_10_ RT versus log_10_ flow rate (log_10_ FR) of each single COS separated by HPAEC on a CarboPacPA-100 column.^a^

Entry	Compound	Linear equation^b^	*R* ^2^
1	D-glucose	*y* = −0.9861*x* + 0.2611	1.0000
2	(COS)_2_	*y* = −0.9894*x* + 0.3314	1.0000
3	(COS)_3_	*y* = −0.9870*x* + 0.5133	1.0000
4	(COS)_4_	*y* = −0.9791*x* + 0.6612	0.9999
5	(COS)_5_	*y* = −0.9789*x* + 0.8380	0.9998
6	(COS)_6_	*y* = −0.9796*x* + 1.0545	0.9998

^a^The mobile phase was A (water), B (0.2 M sodium hydroxide), and C (0.5 M sodium acetate) with the volume ratio of 50 : 30 : 20; ^b^*y* and *x* refer to the log_10_ RT and Log_10_ FR, respectively. The test flow rate range was 0.2, 0.4, 0.6, 0.8, and 1.0 mL/min.

**Table 3:** Linearity of the calibration curve of D-glucose and (COS)_2–6_ standards.

Compound	Linear range (mg/L)	Calibration curve^a^	*R* ^2^	LOD (mg/L)	LOQ (mg/L)
D-glucose	0.3–10.0	*y* = 8.0527*x* + 1.0206	0.9992	0.01	0.03
(COS)_2_	0.3–9.5	*y* = 7.3094*x* + 0.8042	0.9997	0.01	0.03
(COS)_3_	0.3–10.3	*y* = 5.8296*x* + 0.4583	1.0000	0.01	0.03
(COS)_4_	0.3–10.6	*y* = 4.5865*x* + 0.0999	1.0000	0.02	0.05
(COS)_5_	0.3–9.7	*y* = 3.4583*x* + 0.2452	0.9998	0.03	0.09
(COS)_6_	0.4–11.4	*y* = 2.4034*x* + 0.4145	0.9995	0.03	0.11

^a^ *y* and *x* refer to the signal response (nC) and mass concentration (mg/L), respectively. LOD: limit of detection; LOQ: limit of quantification.

**Table 4:** Determination of method precision under repeatability (intraday) and intermediate precision (interday) conditions given as RSD (%) of peak area and retention time.

Analyte	Repeatability (*n* = 7)						Intermediate precision (*n* = 9)					
	Peak area			Retention time			Peak area			Retention time		
	C1	C2	C3	C1	C2	C3	C1	C2	C3	C1	C2	C3
D-glucose	3.83	4.01	3.38	0.08	0.05	0.06	4.84	4.85	3.85	0.68	0.42	0.58
(COS)_2_	3.89	3.22	1.77	0.33	0.30	0.23	3.09	4.32	2.98	0.99	0.85	1.04
(COS)_3_	3.05	2.35	1.52	0.23	0.19	0.28	4.16	3.85	2.87	1.08	0.94	0.98
(COS)_4_	2.69	3.27	1.64	0.21	0.13	0.19	2.51	3.96	3.02	1.12	0.84	0.45
(COS)_5_	3.71	3.65	2.42	0.24	0.12	0.33	4.04	4.02	2.98	1.28	0.65	0.66
(COS)_6_	2.82	3.37	2.41	0.29	0.31	0.36	3.69	4.13	5.11	1.02	1.14	0.75

RSD: relative standard deviation. C1 (mg/L): 1.7; C2 (mg/L): 3.3; C3 (mg/L): 6.7.

**Table 5:** Determination of method accuracy given as recovery using the standard addition method.

Analyte	Recovery (%)		
	Spiked C1	Spiked C2	Spiked C3
D-glucose	90.18 ± 3.53	98.78 ± 0.86	96.68 ± 2.75
(COS)_2_	92.38 ± 1.95	96.96 ± 2.48	102.98 ± 1.58
(COS)_3_	94.32 ± 1.58	97.98 ± 0.56	98.32 ± 3.08
(COS)_4_	92.45 ± 0.87	104.89 ± 2.38	97.42 ± 2.25
(COS)_5_	95.86 ± 0.75	96.28 ± 2.08	102.92 ± 1.88
(COS)_6_	105.18 ± 2.56	96.19 ± 0.64	97.48 ± 2.86

All values were given as mean recovery (*n* = 3) ± standard deviation; C1 (mg/L): 1.7; C2 (mg/L): 3.3; C3 (mg/L): 6.7.
